# Preparation and characterization of a degradable magnesium phosphate bone cement

**DOI:** 10.1093/rb/rbw024

**Published:** 2016-06-28

**Authors:** Ying Yu, Chao Xu, Honglian Dai

**Affiliations:** ^1^State Key Laboratory of Advanced Technology for Materials Synthesis and Processing, Wuhan University of Technology, Wuhan 430070, PR China; ^2^Biomedical Materials and Engineering Research Center of Hubei Province, Wuhan 430070, PR China

**Keywords:** magnesium phosphate bone cement, degradable, biocompatibility

## Abstract

A kind of degradable magnesium phosphate bone cement (MPBC) was fabricated by using the mixed powders of magnesium oxide (MgO), potassium dihydrogen phosphate (KH_2_PO_4_) and calcium dihydrogen phosphate (Ca(H_2_PO_4_)_2_.H_2_O). As MgKPO_4_, the main product of MgO and KH_2_PO_4_ was alkaline, the Ca(H_2_PO_4_)_2_.H_2_O was added to neutralize the alkali of the system. And the effects of Ca(H_2_PO_4_)_2_.H_2_O on the performance of MPBC were discussed. The results showed that the adding of Ca(H_2_PO_4_)_2_.H_2_O extended the setting time, which was about 6 min to 18 min. The compressive strength increased first and then decreased, and maximum value reached 31.2 MPa after setting for 24 h without any additional pressure. The MPBC was degradable in Tris–HCl solution, and the extracts of the cytotoxicity assay showed that the MPBC had good biocompatibility, indicating that the MPBC had good biodegradable and biocompatible properties.

## Introduction

Magnesium phosphate bone cement (MPBC) has attracted much attention in bone regeneration for its high initial strength, fast setting time and moderate degradation rate compared with calcium phosphate bone cement (CMPC) [1–3]. Traditional MPBC is mixed with dead burnt magnesium oxide (MgO) and ammonium dihydrogen phosphate (NH_4_H_2_PO_4_) or diammonium phosphate ((NH_4_)_2_HPO_4_) as solid component, and the main reaction product is magnesium ammonium phosphate hexahydrate (MgNH_4_PO_4_·6H_2_O), known as struvite, a naturally existing crystal [4]. While the problem is that after or during setting they may release ammonia, which may cause cytotoxicity in the physiological environment [5]. However, it has been reported that Mg^2+ ^irons released *in vivo* could increase osteoblast activity, meaning Mg^2+ ^irons play an important role in bone regeneration [6–7]. Therefore, a study by Mestres and Ginebra is focused on the replacements of NH_4_H_2_PO_4_ and (NH_4_)_2_HPO_4_ [8]. On the other hand, potassium dihydrogen phosphate (KH_2_PO_4_) is used to replace NH_4_H_2_PO_4_ in civil engineering, which is called as magnesium potassium phosphate cement (MPP) [9–14]. When compared to NH_4_H_2_PO_4_, KH_2_PO_4_ not only has smaller dissociation constant and lower solubility, resulted in controlling the reaction rate easier, but also does not produce unpleasant odour when reacts with water [15–16], and the final product is magnesium potassium phosphate hexahydrate (MgKPO_4_·6H_2_O), which is isostructural with struvite and also a naturally existing mineral known as struvite-(K) [17–18]. Many researchers studied the engineering properties and reaction mechanisms of MPP [9,19–23]. However, few researches were about applications in MPP for bone repair. In this work, the alkali of MgKPO_4_·6H_2_O is discussed first, which had an adverse effect on the biocompatibility. And then Ca(H_2_PO_4_)_2_ was added into the mixture of MgO and KH_2_PO_4_ to neutralize alkaline of MgKPO_4_·6H_2_O. And the influences of Ca(H_2_PO_4_)_2_ on setting time, compressive strength, degradation rate and biocompatibility are also discussed.

## Materials and Methods

### Preparation of MPBC

MPBC composed of powders and cement liquid (deionized water), and the powders were composed of MgO, KH_2_PO_4_ and Ca(H_2_PO_4_)_2_.H_2_O. The MgO was heated to 1600 °C for 1.5 h, and the particles were grounded to be smaller than 2.68 μm. The KH_2_PO_4_ and Ca(H_2_PO_4_)_2_.H_2_O were grounded and followed by sieving 140 mesh and 200 mesh, respectively. The biocement paste was formed by mixing MPBC powders with deionized water at a powder/liquid ratio of 1.8 g/ml, and placed into plexiglass moulds (size ф10х10 mm) with no added pressure. After storing in water bath with the temperature at 37 °C and 100% relative humidity for 24 h, the hardened MPBC samples were obtained.

The MPBC sample hardened for 24 h was characterized by X-ray diffraction (XRD; Rigaku Co., Japan) and the surface morphology/microstructure was examined by SEM (Zeiss Ultra Plus).

In order to test the pH of MgKPO_4_·6H_2_O, four different mass ratio of MgO and KH_2_PO_4,_ which were 1:3, 1:3.4, 1:3.8, 1:4, were chosen to synthesise MgKPO_4_·6H_2_O. And when the ratio of MgO and KH_2_PO_4_ was 1:3, the Ca(H_2_PO_4_)_2_ was added in and its effects on the performance of MPBC are discussed.

### The pH determination of MPBC extracts

In order to determine the pH in MPBC extracts, hardened samples were soaked in physiological saline solution at a powder/liquid ratio of 0.2 g/ml in tubes and shanked in a temperature oscillation box for 24 h. Then the supernatant was taken out and tested by pH meter.

### Setting time and compressive strength

The setting time of MPBC was measured with a Vicat apparatus bearing a movable rod, weighing 300 g, and a 1 mm needle. The setting time was the number of minutes elapsed from the start of mixing to the time that the needle failed to penetrate more than 1 mm into MPBC paste. The average value was calculated with three tests.

After hardening for 24 h, the compressive strength was measured by a universal testing machine (MTS810, America) with a loading rate of 2 mm/min. Five samples were carried out for each group.

### Degradation in Tris-HCl solution

The degradation of MPBC in Tris-HCl solution (pH = 7.4) was determined by the weight loss ratio at different time points. The hardened samples (ф10х10 mm) were first dried at 50 °C [24] for 2 h, and the initial weight W_0_, was recorded. Then, they were immersed in Tris-HCl solution in a temperature oscillation box at 37 °C and at a liquid/solid mass ratio of 20 ml/g. Then the solution was refreshed every 2 days. After soaking, the specimens were removed from the liquid, rinsed with distilled water and dried at 50 °C for 2 h. And the new weight W_t_ was also recorded. All the values presented were averages of three tests. The weight loss ratio was calculated as follows:

$$\text{Weight loss ratios }=\;({\text{W}}_{0}-W_{t})/{\text{W}}_{0}{}_{\;}\times_{\;}\text{1}00\%$$

### Cell culture

Fibroblast cells (L929) were chosen. They were cultured in complete Roswell Park Memorial Institute 1640 (RPMI) containing 10% foetal bovine serum (FBS), 1% antibiotics (penicillin, streptomycin) and at 37°C in a humidified incubator with 5% CO_2_. The cells were harvested at confluence with 0.25% trypsin and seeded onto the disks, respectively, with an initial density of 2,000 cells per well in a 96-well plate and incubated at 37°C/CO_2_. The medium was replaced every 3 days.

### The cytotoxicity of MPBC

The extracts of MPBC were prepared according to the procedures reported in the literature [25]. Firstly, the solutions were obtained by adding sterilized powders into serum-free RPMI at a solid/liquid ratio of 0.2 g/ml. After incubation at 37 °C for 24 h, the mixture was centrifuged, and the supernatant was collected and then stored at 4 °C for further use.

The cytotoxicity of MPBC was evaluated by an MTT (3-(4,5-dimethylthiazol-2yl)-2,5-disphenyl-2H-tetrazolium-bromide) assay at day 1, day 3 and day 5. A 20 μl MTT solution (5 mg ml ^−^ ^1^) was added into each well and incubated for 4 h at 37°C/5% CO_2_. Then, the medium was discarded and 200 μl dimethylsulfoxide (DMSO) was added to dissolve purple crystals. The optical density (OD) of the solution was tested by a microplate reader on the first, third and fifth day at 490 nm.

## Results and Discussion

### The pH of KMgPO_4_

**Figure 1.**
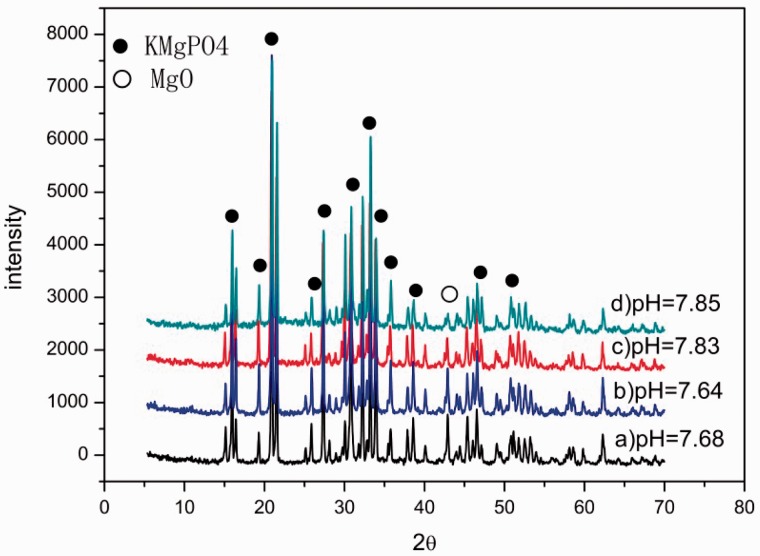
the XRD and pH results. a) 1:3; b) 1:3.4; c) 1:3.8; d)1:4.

Figure 1 shows the XRD and pH results when the MgO and KH_2_PO_4_ were at different mass ratios. The formula of X-ray diffraction intensity is shown in 3-1. It meant that the XRD diffraction intensity was not linear with the content of the phase. But when the samples were formed by substances possessing the same structure, it could result in the same parameters in 3-1. In this particular case, the intensity of the diffraction peak would increase with the increase of the phase content. All the samples were composed of MgO and KMgPO_4_ (Fig. 1). The diffraction peak intensity of MgO decreased, while the diffraction peak intensity of KMgPO_4_ increased with the increase of KH_2_PO_4_, meaning that MgO was totally transformed into KMgPO_4_. And the pH got larger at the same time, proving that the KMgPO_4_ was alkaline.

3-1 $$\text{I}=\text{M}\cdot \text{Lp}\cdot {\text{F}}^{2}\cdot e^{-\text{2M}}\cdot \text{A}*\left(\theta \right)\;\cdot \left(\text{PO}\right)$$

M—Multiple factors, Lp—Lorentz polarization factor, F^2^—Structural factor, e^−2M^—Temperature factor, A*(θ)—Absorption factor, PO—Preferred orientation factor.

### Measurement of setting time and compressive strength

**Figure 2.**
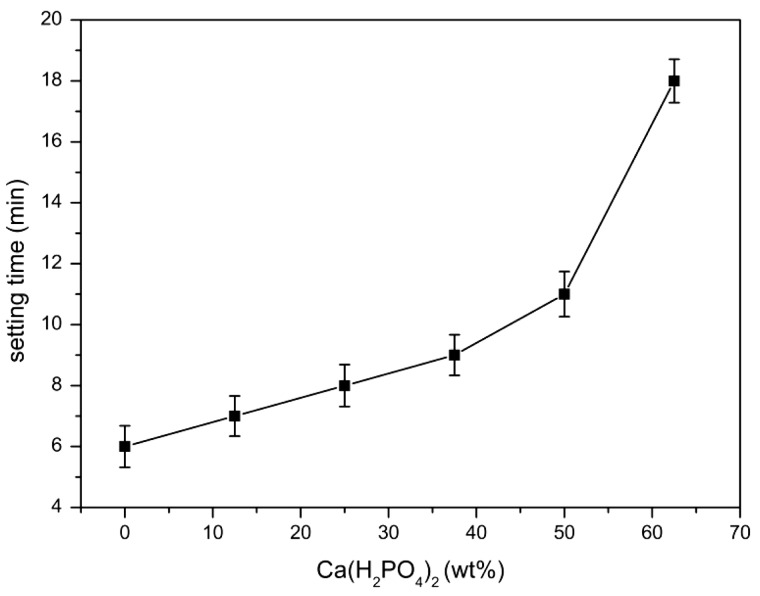
Effect of Ca(H_2_PO_4_)_2_ on the setting time.

**Figure 3.**
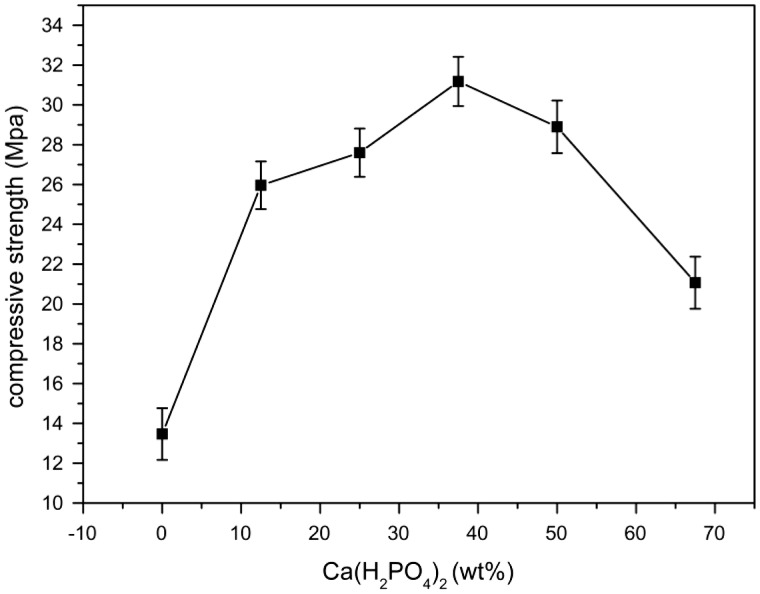
Effect of Ca(H_2_PO_4_)_2_ on the compressive strength.

**Figure 4.**
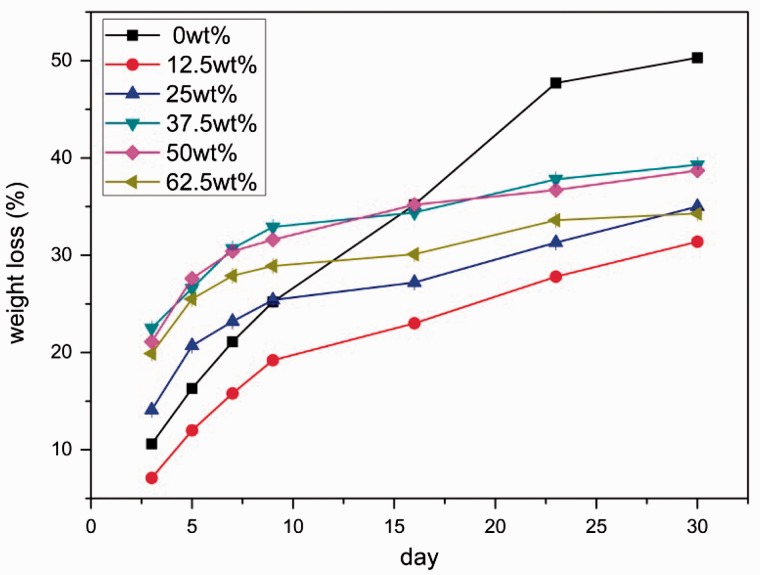
the SEM images of MPBC samples (1)0% Ca(H_2_PO_4_)_2_ (2) 12.5% Ca(H_2_PO_4_)_2_ (3) 25% Ca(H_2_PO_4_)_2_ (4) 37.5% Ca(H_2_PO_4_)_2_ (5)50% Ca(H_2_PO_4_)_2_ (6) 62.5% Ca(H_2_PO_4_)_2_.

The effect of Ca(H_2_PO_4_)_2_ on the setting time is shown in Fig. 2. The setting time of MPBC increased with an increase content of Ca(H_2_PO_4_)_2_. When the ratio of MgO and KH_2_PO_4_ was 1:3, the content of MgO was excessive to the reaction (Table 2). While with the increase of Ca(H_2_PO_4_)_2_, the acidity of the reaction system was increased, producing more crystalline products, and the main product KMgPO_4_ disappeared, which extended the time to reach the equilibrium of the reaction, leading longer setting time. Figure 3 shows the effect of Ca(H_2_PO_4_)_2_ on the compressive strength. The compressive strength increased with the increase of Ca(H_2_PO_4_)_2_, and reached a maximum value of 31.2Mpa after setting for 24 h without additional pressure, higher than the traditional NH_4_-MPC, 29Mpa with added pressure [26]. The increasing compressive strength was owing to the increase of acidity in the system, resulting in more clay-like substances, and they stacked together to form the strength (Fig. 4). Yet the decreasing of the compressive strength was because that the degree of crystallinity about the products was reduced, and the structure also became looser (Fig. 4), resulted in decreasing of compressive strength.

**Table 1.** XRD results of MPBC after setting for 24 h and degraded for 30 d. (1) 0 wt% (2)12.5 wt% (3)25 wt% (4)37.5 wt%(5)50 wt% (6)62.5 wt%

	1	2	3	4	5	6
Setting for 24 h	①②	②③④⑤	②④⑤⑥⑦⑧	②④⑤⑥⑦⑧⑨	⑤⑥⑦⑧⑨	⑤⑥⑦⑧⑨
Degraded for 30 d	❷❺❻❼	❶❸❽❾	❶❷❸❿ 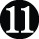	❶❷ 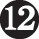	❶❷❽	❶❷❽

①MgO②KMgPO_4_③CaKPO_4_④Ca_3_(PO_4_)_2_⑤Mg_3_(PO_4_)_2_⑥MgHPO_4_⑦MgCa_2_(PO_4_)_2_⑧HA.

⑨MgKH(PO_4_)_2_.

❶MgHPO_4_❷Mg_3_(PO_4_)_2_❸KMgPO_4_ ❺MgH_2_P_2_O_7_❻Mg_2_P_2_O_7_❼K_4_P_2_O_7_❽K_2_CaP_2_O_7_❾CaKPO_4_❿Mg_2_KH(PO_4_)_2_
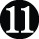
Ca_3_(PO_4_)_2_
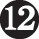
K_2_CaH_4_(P_2_O_7_)_2_.

**Table 2.** RGR and toxicity grade conversion

Toxicity Grade	RGR (%)
Grade 0	≥100
Grade 1	75-99
Grade 2	50-74
Grade 3	25-49
Grade 4	1-24
Grade 5	0

### XRD analysis and degradation rate

The phase composition of the hardened MPBC with different content of Ca(H_2_PO_4_)_2_ was characterized by XRD as shown in Table 1. The MPBC contained a mixture of KMgPO_4_ and unreacted MgO when the raw materials were KH_2_PO_4_ and MgO. With the increase of Ca(H_2_PO_4_)_2_, MgO disappeared, and MgHPO_4_, MgCa_2_(PO_4_)_2_, Mg_3_(PO_4_)_2_ and Ca_3_(PO_4_)_2_ emerged. If the Ca(H_2_PO_4_)_2_ was more than 50 wt%, KMgPO_4_ would disappear, and the final composition was MgHPO_4_, Mg_3_(PO_4_)_2_, MgCa_2_(PO_4_)_2_ and MgKH(PO_4_)_2_, which was in accordance with the results of the pH in MPBC extracts.

Figure 3 presents the weight loss ratio of MPBC samples immersed in Tris-HCl solution at different time. Clearly, the MPBC degraded in Tris-HCl solution with time, and the degradation rate was related to the amount of products as shown in Table 2. From Table 2, we could see that the amount of products after degradation for 30 days decreased the most was the MPBC sample with 37.5 wt% Ca(H_2_PO_4_)_2_. And it had the fastest degradation rate, which meant the more the products disappeared, the fast the degradation rate was.

After the degradation of 30 d, the MgO and KMgPO_4_ changed into magnesium phosphate (Mg_3_(PO_4_)_2_), magnesium dihydrogen pyrophosphate (MgH_2_P_2_O_7_), magnesium pyrophosphate (Mg_2_P_2_O_7_) and potassium pyrophosphate (K_4_P_2_O_7_). The MPBC samples with cement powder containing Ca(H_2_PO_4_)_2_ would produce magnesium hydrogen phosphate (MgHPO_4_) after setting for 24 h (Table 1). And after the degradation of 30 d, there were also MgHPO_4_ emerged. While the other products changed into pyrophosphate.

### The pH of MPBC extracts

**Figure 5.**
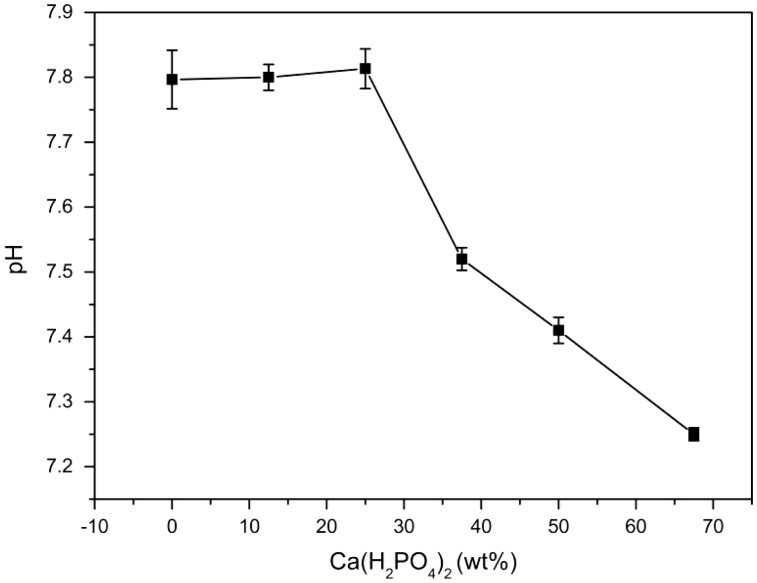
Effect of Ca(H_2_PO_4_)_2_ on the degradation rate.

The result of pH in MPBC extracts is shown in Fig. 5. The pH decreased with the increased content of Ca(H_2_PO_4_)_2_, related to the products after setting for 24 h. With the increase of Ca(H_2_PO_4_)_2_, both KMgPO_4_ and MgO disappeared, and MgHPO_4_ emerged, causing the decrease in pH of MPBC extracts, which could also prove the alkali of KMgPO_4_.

### The SEM images of MPBC samples

Figure 4 presents SEM images of the surface morphology/microstructure of MPBC with different content of Ca(H_2_PO_4_)_2_ after setting for 24 h. It was found that the MPBC without Ca(H_2_PO_4_)_2_ contained cylinder-like crystals which should be the KMgPO_4_ and clay-like substances to form a dense structure, and the crystals were in close proximity to the clay-like substances as shown in Fig. 4(1). With the increase of Ca(H_2_PO_4_)_2_, the cylinder-like crystals disappeared while the clay-like substances grown, which was corresponding to the XRD results that the KMgPO_4_ would disappear with the increase of Ca(H_2_PO_4_)_2_. And we could see that the clay-like substances stacked together to form the strength of the sample from Fig. 4(2) to Fig. 4(4). And also there were many small voids on the clay-like substances. When the content of Ca(H_2_PO_4_)_2_ was more than 50%, the clay-like substances were grown to smooth surface with loose and porous structure under it, which caused the decrease of the MPBC sample.

### The cytotoxicity of MPBC

The cytotoxicity of the MPBC was investigated through MTT assay. The MTT assay is very often used to evaluate cell proliferation and the viability for biomaterial toxicity. In this experiment, the extracts of the MPBC samples were adjusted to examine the cytotoxicity on L929 cells.

**Figure 6.**
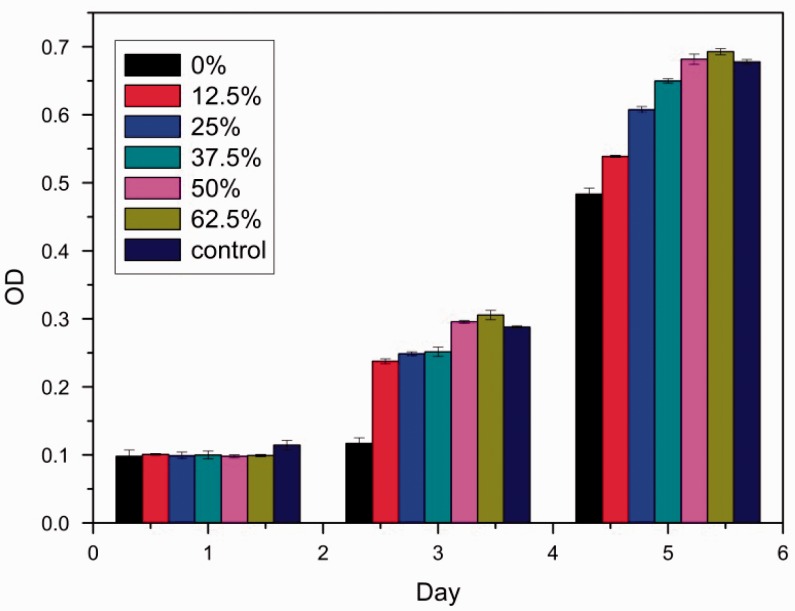
results of cytotoxicity of MPBC samples.

**Figure 7.**
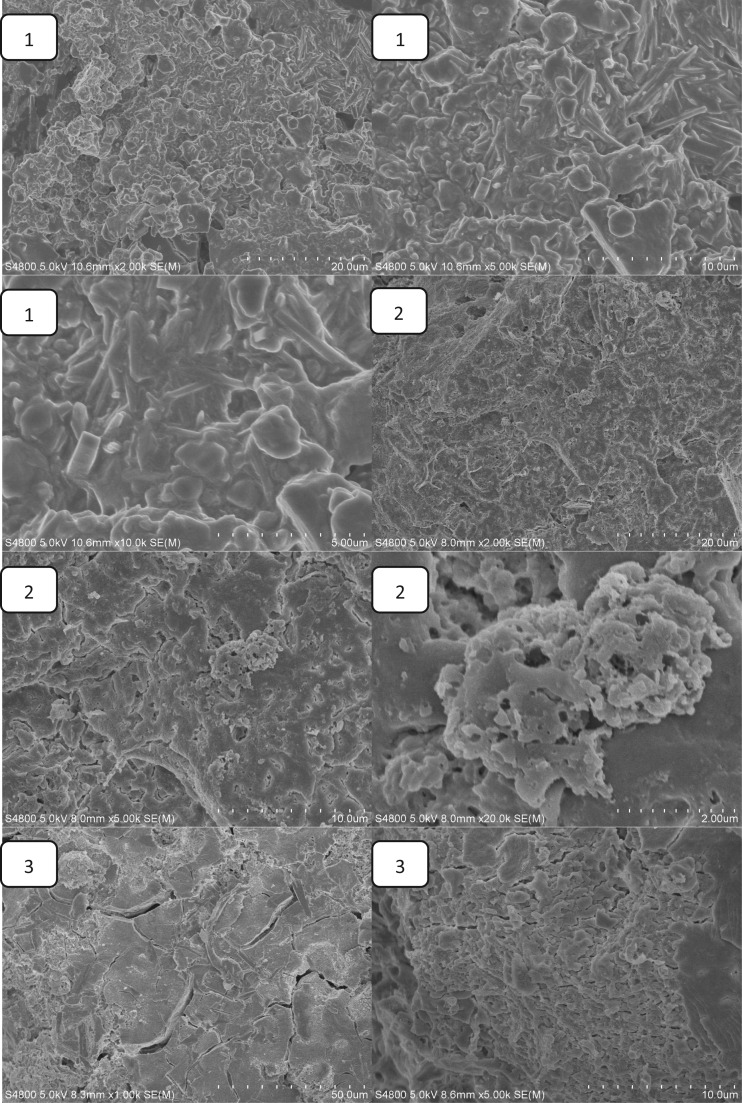

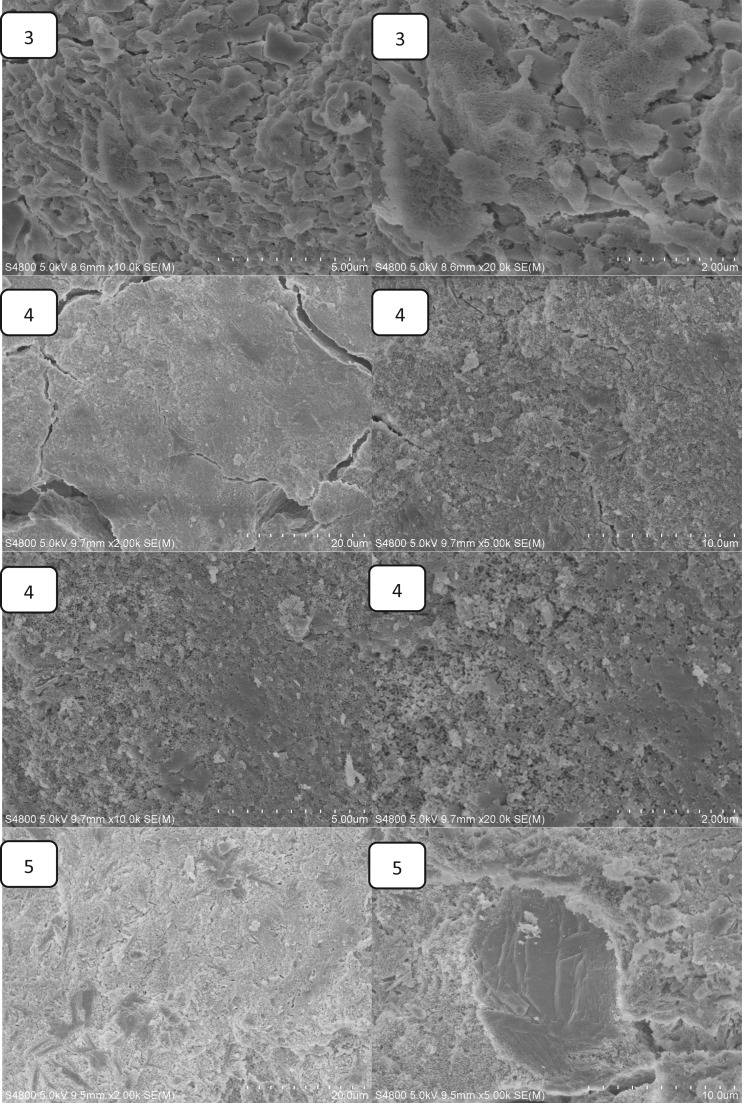

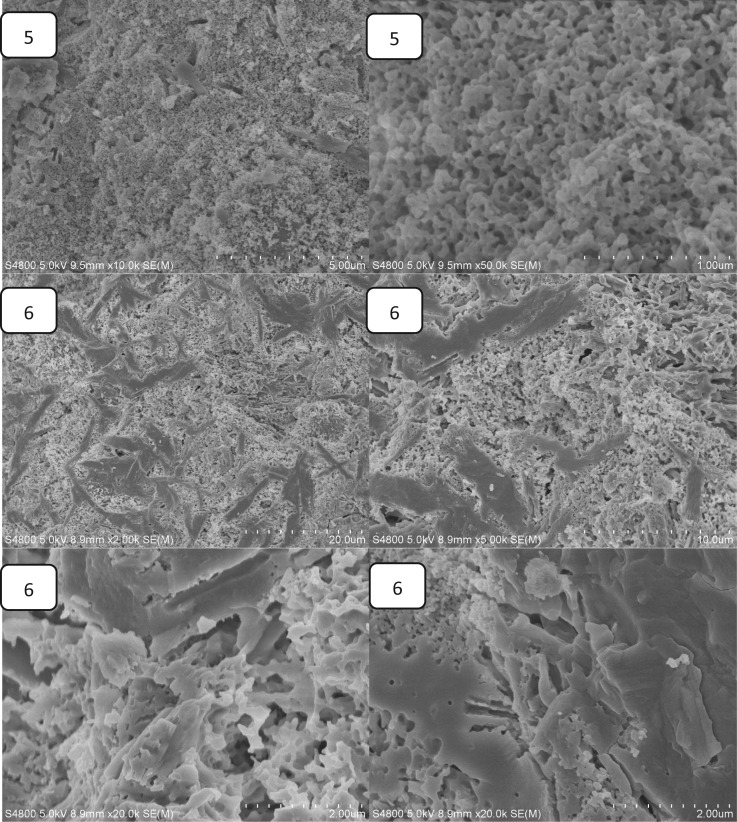
Effect of Ca(H_2_PO_4_)_2_ on the pH in MPBC extracts.

According to RGR and toxicity grade conversion table (Table 1), the toxicity of MPBC samples when the content of Ca(H_2_PO_4_)_2_ was less than 50% were classified as grade 1, and the other samples were classified as grade 0, suggesting that they have good biocompatibility for cellular application (Fig. 6). It was because that the addition of Ca(H_2_PO_4_)_2_ reduced the pH in MPBC extracts (Fig. 7), which could also prove that the alkaline KMgPO_4_ had some bad effects on the biocompatibility.

## Conclusion

A novel of degradable MPBC was developed by using a mixture of MgO, KH_2_PO_4_ and Ca(H_2_PO_4_)_2_.H_2_O as cement powders. With the increase of Ca(H_2_PO_4_)_2_, the setting time extended, and the range was 6 min to 18 min. While the compressive strength increased first and then decreased, and the maximum value could reach to 31.2Mpa after setting for 24 h. The MPBC was degradable in Tris-HCl, and the cytotoxicity assays of MPBC extracts showed the good biocompatibility of the samples, suggesting the promise of the MPBC as a bioactive biomaterial for bone regeneration.
